# Ebola virus requires a host scramblase for externalization of phosphatidylserine on the surface of viral particles

**DOI:** 10.1371/journal.ppat.1006848

**Published:** 2018-01-16

**Authors:** Asuka Nanbo, Junki Maruyama, Masaki Imai, Michiko Ujie, Yoichiro Fujioka, Shinya Nishide, Ayato Takada, Yusuke Ohba, Yoshihiro Kawaoka

**Affiliations:** 1 Department of Cell Physiology, Faculty of Medicine and Graduate School of Medicine, Hokkaido University, Sapporo, Hokkaido, Japan; 2 Division of Global Epidemiology, Hokkaido University Research Center for Zoonosis Control, Sapporo, Hokkaido, Japan; 3 Division of Virology, Department of Microbiology and Immunology, Institute of Medical Science, University of Tokyo, Tokyo, Japan; 4 Global Station for Zoonosis Control, Global Institution for Collaborative Research and Education, Hokkaido University, Sapporo, Hokkaido, Japan; 5 Department of Pathobiological Sciences, School of Veterinary Medicine, University of Wisconsin-Madison, Madison, Wisconsin, United States of America; 6 Department of Special Pathogens, International Research Center for Infectious Diseases, Institute of Medical Science, University of Tokyo, Tokyo, Japan; University of Texas Medical Branch, UNITED STATES

## Abstract

Cell surface receptors for phosphatidylserine contribute to the entry of Ebola virus (EBOV) particles, indicating that the presence of phosphatidylserine in the envelope of EBOV is important for the internalization of EBOV particles. Phosphatidylserine is typically distributed in the inner layer of the plasma membrane in normal cells. Progeny virions bud from the plasma membrane of infected cells, suggesting that phosphatidylserine is likely flipped to the outer leaflet of the plasma membrane in infected cells for EBOV virions to acquire it. Currently, the intracellular dynamics of phosphatidylserine during EBOV infection are poorly understood. Here, we explored the role of XK-related protein (Xkr) 8, which is a scramblase responsible for exposure of phosphatidylserine in the plasma membrane of apoptotic cells, to understand its significance in phosphatidylserine-dependent entry of EBOV. We found that Xkr8 and transiently expressed EBOV glycoprotein GP often co-localized in intracellular vesicles and the plasma membrane. We also found that co-expression of GP and viral major matrix protein VP40 promoted incorporation of Xkr8 into ebolavirus-like particles (VLPs) and exposure of phosphatidylserine on their surface, although only a limited amount of phosphatidylserine was exposed on the surface of the cells expressing GP and/or VP40. Downregulating Xkr8 or blocking caspase-mediated Xkr8 activation did not affect VLP production, but they reduced the amount of phosphatidylserine on the VLPs and their uptake in recipient cells. Taken together, our findings indicate that Xkr8 is trafficked to budding sites *via* GP-containing vesicles, is incorporated into VLPs, and then promote the entry of the released EBOV to cells in a phosphatidylserine-dependent manner.

## Author summary

Although Ebola virus causes severe hemorrhagic fever with a high mortality rate, there are no approved therapeutics. The viral entry process is one of the targets for antiviral development. Previous studies suggest that binding of phosphatidylserine, a component of the viral envelop, to the receptors promotes the entry of Ebola virus. Ebola virus is released from the surface membrane of infected cells. However, phosphatidylserine normally distributes in the inner layer of the cell surface membrane, suggesting that phosphatidylserine is likely flipped to the outer leaflet of the membrane in infected cells for Ebola virus to acquire it. Because the mechanism by which phosphatidylserine changes its orientation in Ebola virus-infected cells is poorly understood, we studied and identified a cellular enzyme, XK-related protein 8 (Xkr8), as a responsible factor involved in this process. We demonstrated that the Ebola virus glycoprotein promoted the incorporation of Xkr8 in viral particles, which flips phosphatidylserine on their surface, enhancing their entry to cells. Our findings provide new insights into the mechanism of Ebola virus infection, which may be exploited for the development of therapeutics against Ebola virus infection.

## Introduction

Ebola virus (EBOV), a member of the family *Filoviridae*, is an enveloped, single-stranded, negative-sense RNA virus that causes severe hemorrhagic fever with a high mortality rate in humans and nonhuman primates [1]. Currently, there are no FDA-approved therapeutics to treat EBOV infection [2]. The viral entry process is one of the targets for antiviral development. Several early studies investigated the mechanism by which EBOV enters host cells, focusing on the interaction of GP with cell surface attachment factors. EBOV entry is initiated by the binding of GP to attachment factors (e.g., C-type lectins), which induces macropinocytosis-mediated uptake of the virions [3–5]. The internalized virions are then trafficked in the endosomal pathway, during which GP is processed by cathepsins in low pH-dependent manner. Proteolysis of GP allows the putative receptor-binding region to be exposed and subsequently interact with a cholesterol transporter, Niemann-Pick C1 (NPC1), which initiates fusion of the viral envelope with the endosomal membrane [6–8].

Recent accumulating evidence supports the importance of cellular phospholipids in the viral life cycle. Specifically, various viruses, including EBOV, exploit apoptotic mimicry to facilitate their entry by acquiring phosphatidylserine (PS) on the external leaflet of their envelopes [9–21]. The PS exposed on the surface of virions initiates viral uptake by binding to its receptors, such as T-cell immunoglobulin and mucin domain (TIM) [16, 18] and TYRO3, AXL, and the MERTK family of receptor tyrosine kinases (TAMs) family [15, 16, 19–22].

Ectopic expression of TIM or TAMs has been shown to enhance macropinocytosis-mediated uptake of EBOV viral-like particles (Ebola VLPs) and psudovirions possessing GP [3, 12, 21, 22]. Uptake of EBOV particles by recipient cells was inhibited in the presence of liposomes containing PS and annexin V [11, 18], suggesting that externalized PS likely in the viral envelope is important for the binding of viral particles to TIM-1 and subsequent entry, in addition to GP binding to cell surface moieties.

Currently, the mechanisms underlying how PS is externalized on the outer leaflet of the viral envelope are poorly understood. PS, like other lipid species, is distributed heterogeneously throughout the cell. PS is synthesized in the cytosolic leaflet of the ER and transported *via* vesicular transport to the plasma membrane (PM) through the secretory pathway. It is initially enriched in the luminal leaflet of the endoplasmic reticulum (ER) and *cis*-Golgi [23], and then flipped to the cytosolic leaflet in the *trans*-Golgi network (TGN) and the subsequently generated secretory vesicles [24]. These vesicles conserve the topology of PS when they fuse to the PM, resulting in exclusive distribution of PS in the inner leaflet of the PM [25]. Three types of enzymes are involved in the formation and disruption of membrane asymmetry: flippases, floppases, and scramblases [26]. Flippases are type IV P-type ATPases (P4-ATPases) that are involved in the formation of membrane asymmetry by flipping specific phospholipid species from the extracellular leaflet to the cytosolic side. Floppases are ABC transporters that mediate the translocation of phospholipids in the reverse direction. Scramblases disrupt lipid asymmetry through random bidirectional translocation of phospholipids between the leaflets in an ATP-independent manner. Apoptotic stimuli initiate both activation of the scramblase Xkr8 [27, 28] and inactivation of the flippase ATP11C [29], which leads to irreversible disruption of the asymmetrical distribution of PS in the PM. Extracellular PS then serve as an ‘eat me’ signal, which is recognized by PS receptors on phagocytes, removing the apoptotic cells.

In our present study, we characterize the role of the scramblase Xkr8 in the externalization of PS on the surface of Ebola VLPs to understand the mechanism underlying PS-dependent EBOV entry. The XK-related family possesses six putative transmembrane domains. Of the nine murine members of the XK-related family, Xkr8 is ubiquitously expressed [30]. Its *C*. *elegans* homolog, CED-8, has been implicated in the exposure of PS during programmed cell death [27, 31]. Xkr8 contains a caspase-recognition site in its C-terminus. Upon apoptotic stimuli, Xkr8 is activated by cleavage with a caspase and mediates the scrambling of PS as well as that of phosphatidylethanolamine (PE), which results in their exposure on the outer leaflet of the PM.

Here, we demonstrate that co-expression of the major matrix protein VP40 and GP promotes trafficking of Xkr8 to budding sites in the PM *via* GP-positive vesicles and that Xkr8 is incorporated into Ebola VLPs. A fraction of the incorporated Xkr8 is cleaved in a caspase-dependent manner and mediates exposure of PS on the outer leaflet of the envelope of the virions. Taken together, our findings provide new insights into GP’s function in EBOV entry. Since disrupting the externalization of PS on the virions suppressed EBOV entry, this pathway is a potential target for the development of therapeutics for the treatment of EBOV infection.

## Results

### PS is externalized on the surface of Ebola VLPs

**Fig 1:**
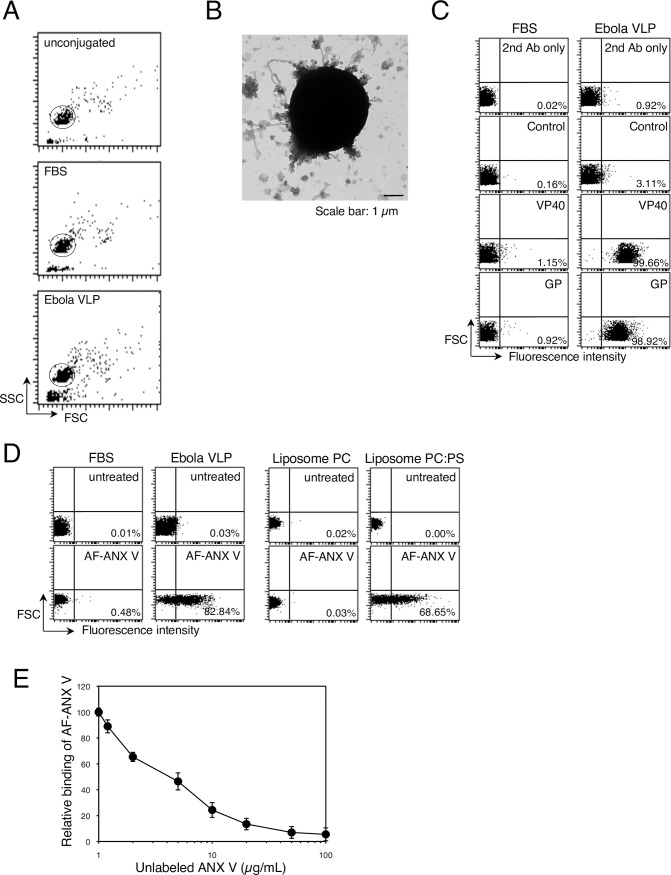
Externalization of PS on the surface of Ebola VLPs. (A) Representative dot plots of unconjugated (top), FBS- (middle), or Ebola VLP- (bottom) conjugated beads. Purified Ebola VLPs or FBS were incubated with 4-μm latex beads and then analyzed by means of flow cytometry. Single beads (circle) were gated for further analysis. X-axis: forward scatter corner signals, Y-axis: side scatter corner signals. (B) Electron micrograph of Ebola VLP-conjugated beads. Latex beads conjugated with Ebola VLPs were subjected to negative staining. Scale bar: 1 μm. (C) Binding of antibodies to the beads. FBS- (left) or Ebola VLP-conjugated (right) beads were incubated with rabbit polyclonal antibodies against EBOV GP, or VP40, followed by incubation with Alexa Fluor 488-labeled secondary antibody. To analyze the binding of the anti-VP40 antibody, we treated Ebola VLP-conjugated beads with 0.05% Triton X-100 for 10 min at room temperature. Antibody binding to the beads was analyzed by flow cytometry. 2^nd^ Ab indicates beads that were not treated with primary antibody. As a control, the rabbit anti-LASV GPC polyclonal antibody was used. The percentages of the positive populations are indicated. X-axis: fluorescent intensity, Y-axis: forward scatter corner signals showing the size of the gated events. The results are representative of three individual experiments. (D) Binding of fluorescently labeled-ANX V to the beads. FBS- (left) or Ebola VLP-conjugated (right) beads were incubated with or without Alexa Fluor 488 (AF)-ANX V. The percentages of the positive populations are indicated. X-axis: fluorescent intensity, Y-axis: forward scatter corner signals. The results are representative of three individual experiments. (E) The effect of unlabeled ANX V on the binding of AF-ANX V to the beads. Ebola VLP-conjugated beads were pretreated with unlabeled ANX-V at the indicated concentrations. After being washed, the beads were incubated with 1.2 μg/ml AF-ANX V for 15 min at room temperature. Binding of AF-ANX V to the beads was analyzed by flow cytometry. Relative binding of AF-ANX V to the beads is shown. Each experiment was performed in triplicate and the results are presented as the mean ± SD.

Several groups, including ours, have reported that uptake of Ebola VLPs into target cells is dependent on TIM family members [11, 13, 18, 32]. We first assessed the presence of PS on the surface of Ebola VLPs by using a flow cytometry-based analysis [33, 34]. It has previously been shown that co-expression of EBOV-encoded major matrix protein VP40 and GP glycoprotein yields VLPs possessing filamentous architecture that resembles authentic EBOV [35–37]. Because direct interaction of VP40 and viral nucleoprotein (NP) is required for nucleocapsid (NC) recruitment into viral particles [38], resulting in efficient VLP production [39], we obtained Ebola VLPs by co-expressing VP40, GP, and NP in HEK293T cells [4]. Ebola VLPs were then conjugated to aldehyde/sulfate latex beads, and incubated with antibodies to characterize the surface molecules by means of flow cytometry [33, 34]. Single beads were gated for further analysis (Fig 1A). We proved that intact VLPs were conjugated with the beads by using electron microscopy (Fig 1B). Filovirus is known to produce filamentous and spherical viral particles [40]. Both filamentous and spherical viral particles were attached to the beads at a 1:4–10 ratio. We also confirmed the presence of intact VLPs by performing a protease protection assay [41] (S1 Fig). Ebola VLP-conjugated beads were treated with trypsin and/or Triton X-100 under at room temperature for 30 min. The antibody against VP40 bound more efficiently to the VLP-conjugated beads permeabilized with Triton X-100 than to untreated beads (Fig 1C and S1 Fig). While treatment with trypsin alone did not allow the anti-VP40 antibody to access its antigen in VLPs, trypsinization in the presence of Triton X-100 significantly abolished the binding of the antibody to the beads, indicating that VP40 is efficiently protected from trypsin in VLPs. The majority of the Ebola VLP-conjugated beads were also specifically recognized by the antibody against EBOV GP (Fig 1C). We also confirmed that Triton X-100 treatment did not affect the binding of the anti-GP antibody to the Ebola VLP conjugated beads (S2 Fig). These results indicate that most of the beads were conjugated with intact VLPs.

We then conducted the latex bead-based analysis using Alexa Fluor 488-labeled Annexin V (AF-ANX V) to determine whether PS was present on the surface of the VLPs. AF-ANX V efficiently bound to Ebola VLP-conjugated beads, but not to control beads (Fig 1D). We confirmed that AF-ANX V bound to the beads conjugated with liposomes consisting of phosphatidylcholine (PC) and PS in the ratio of 1:3, but not to the beads conjugated with liposomes consisting of PC alone. Pre-treatment of the VLP-conjugated beads with unlabeled ANX V blocked the binding of AF-ANX V to the beads in a dose-dependent manner (Fig 1E), confirming the presence of PS on the surface of the Ebola VLPs.

### Xkr8 localizes along with GP in intracellular vesicles

**Fig 2:**
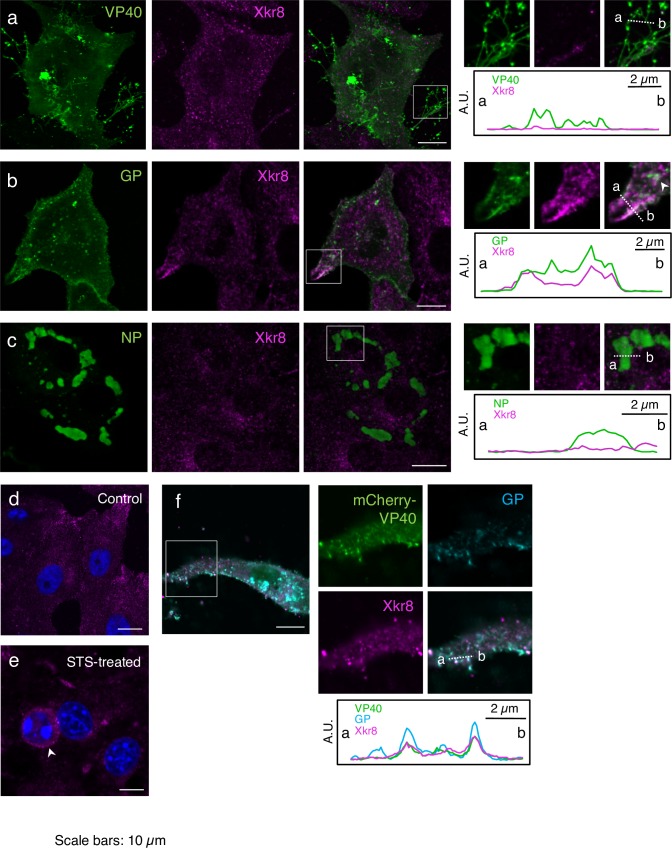
Distribution of Xkr8 in cells expressing EBOV proteins. Vero-E6 cells grown on cover slips were transfected with the expression plasmids of EBOV VP40 (a), GP (b), NP (c), or mCherry-VP40 and wtVP40 (1:5) along with GP (f). At 48 h.p.t. the distribution of the individual EBOV proteins and Xkr8 (magenta) were analyzed by immunofluorescence staining. Viral proteins are shown in green (a–c). In panel (f), mCherry-VP40 and GP are shown in green and cyan, respectively. As a control, a backbone plasmid was transfected (d). Panel (e) represents Vero-E6 cells treated with 1 μM STS for 6 h. Insets show the boxed areas. The plots indicate relative fluorescence intensity detected with individual channels along each of the corresponding lines. A.U.; arbitrary unit. Scale bars in the large panels: 10 μm.

To elucidate the molecular mechanism underlying the externalization of PS on Ebola VLPs, we explored the role of Xkr8. We first examined its intracellular distribution in Vero-E6 cells transiently expressing VP40, GP, or NP individually. In agreement with previous findings [37, 41–44], immunofluorescence staining revealed that VP40 was distributed in multiple subcellular compartments (Fig 2A, green). VP40 was visualized diffusely in the cytoplasm and the nucleus, and particularly in the PM as intense fibrous structures.

GP forms trimeric spikes on virions [45] and plays an essential role in virus entry by mediating receptor binding and fusion [46–48]. Several posttranslational modifications in the ER and Golgi apparatus result in the mature GP product [48–51]. GP is subsequently transported to the PM, where EBOV acquires it while budding from the PM [45]. When GP was expressed alone, it was distributed in the cytoplasm in a vesicle-shaped pattern and in the PM (Fig 2B, green).

NP is a nucleoprotein that associates with the viral genome and assembles into a helical NC along with the viral polymerase cofactor (VP35), the transcription activator (VP30), and the RNA-dependent RNA polymerase (L) [37, 52, 53]. When NP was expressed alone, it formed distinct aggregates in the cytoplasm (Fig 2C, green).

Previous studies indicate that exogenously expressed GFP-fused Xkr8 distributes mainly in the PM and partly in the cytoplasm of HEK293T cells, acute non-lymphocytic leukemia PLB985 cells, and a murine pro-B cell line, Ba/F3 [27, 28, 54]. However, immunofluorescence staining of Vero-E6 cells revealed that most of the endogenous Xkr8 distributed in the cytoplasm as a speckled pattern (Fig 2D), suggesting that it predominantly localizes in intracellular vesicles. We confirmed the typical distribution of endogenous Xkr8 in human cells such as HEK293T cells and in the gastric epithelial NU-GC-3 cell line, and of its exogenously expressed FLAG- and GFP-tagged derivatives in HEK293T cells (S3 Fig). Upon apoptotic stimulation with staurosporine (STS), Xkr8 distribution was not altered, but cells exhibited a round-up morphology typical to such apoptotic cells (Fig 2E, arrow head).

We next characterized the distribution of Xkr8 upon expression of EBOV proteins. Co-immunofluorescent staining revealed that Xkr8 partially localized with GP-positive speckles and their adjacent regions (Fig 2B, white arrow head). We then analyzed the distribution of Xkr8 and GP in Vero-E6 cells expressing GFP-fused to Rab7, a late endosome marker and found that they partially localized together in Rab7-positive endosomes (S4 Fig, inset A), suggesting that Xkr8 is trafficked along with GP in the intracellular vesicles. Some GP signals in the PM also appeared to be co-localized with Xkr8 (Fig 2B and S4 Fig, inset B). In contrast, Xkr8 co-localized with neither VP40 (Fig 2A) nor NP (Fig 2C).

Co-expression of VP40 and GP has been reported to enhance the release of Ebola VLPs [11, 36, 39]. Therefore, we investigated the impact of co-expression of VP40 and GP on the distribution of Xkr8. We transfected Vero-E6 cells with expression plasmids for VP40 fused to a derivative of monomeric red fluorescent protein, mCherry (mCherry-VP40) and wild-type (wt)VP40 in the ratio of 1:5, along with GP, which yielded mCherry-positive fibrous structures at the periphery of the cells (Fig 2F, green). We observed that Xkr8 (Fig 2F, magenta) partly co-localized with VP40 (green) and GP (cyan) at the PM, suggesting that co-expression of VP40 and GP promoted the localization of Xkr8 to the sites where Ebola VLPs bud.

### Co-expression of VP40 and GP promotes the incorporation of Xkr8 into Ebola VLPs

**Fig 3:**
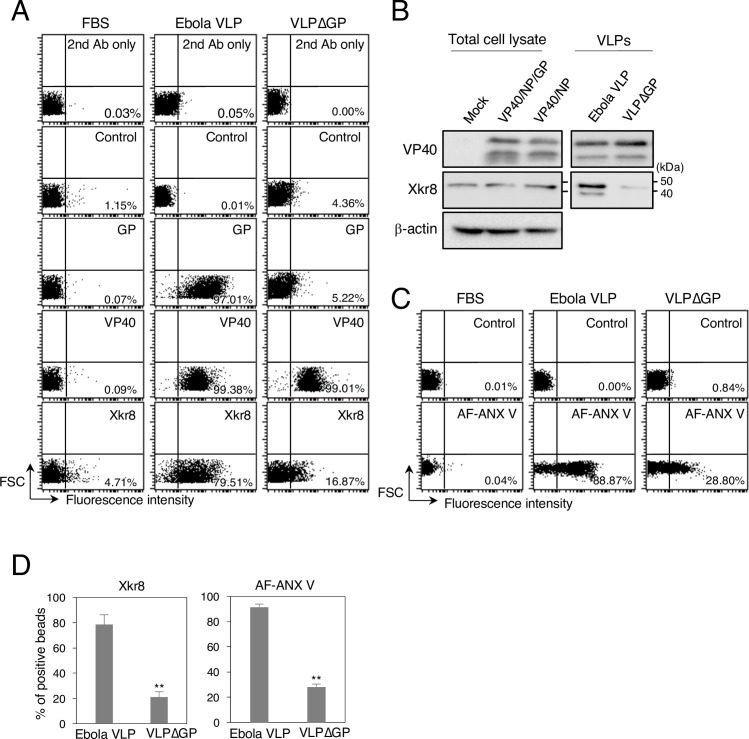
Role of GP in the incorporation of Xkr8 in Ebola VLPs. (A) Characterization of incorporated Xkr8 in Ebola VLPs by means of flow cytometry. FBS- (left), Ebola VLP- (middle), or VLPΔGP- (right) conjugated beads incubated with the rabbit anti-VP40, GP, or Xkr8 polyclonal antibodies. For the binding of antibodies against VP40 and Xkr8, the beads were pre-treated with Triton X-100. 2^nd^ Ab indicates samples that were not treated with primary antibody. As a control, the rabbit anti-LASV GPC polyclonal antibody was used. The percentages of the positive populations are indicated. X-axis: fluorescence intensity, Y-axis: forward scatter corner signals. The results are representative of three individual experiments. (B) Characterization of incorporated Xkr8 in Ebola VLPs by western blotting. 293T cells were transfected with the expression plasmids of VP40 and NP, with or without GP. At 48 h.p.t., the cells and culture medium were harvested. Viral particles were purified from the culture medium. Total cell lysates and VLPs were analyzed by western blotting with the rabbit polyclonal antibodies against VP40, GP, Xkr8, or β-actin. (C) Binding of AF-ANX V to the beads. FBS- (left), Ebola VLP- (middle), or VLPΔGP- (right) conjugated beads were incubated with AF-ANX V. The percentages of the positive populations are indicated. X-axis: fluorescence intensity, Y-axis: forward scatter corner signals. The results are representative of three individual experiments. (D) Summary of the binding of the anti-Xkr8 antibody (left) and AF ANX V (right) to the Ebola VLP- or VLPΔGP-conjugated beads. Each experiment was performed in triplicate and the percentages of the positive populations are presented as the mean ± SD. **, *P* < 0.01 versus respective control (Student’s *t* test).

Because Xkr8 appeared to localize with VP40 and GP in the PM (Fig 2F), we examined whether Xkr8 is incorporated into viral particles by using a latex bead-based analysis. The majority of the Ebola VLP-conjugated beads treated with Triton X-100 were recognized by the antibody against Xkr8 (Fig 3A, middle panels), suggesting that Xkr8 is likely incorporated into viral particles.

VP40 has been shown to associate with the inner layer of the PM through PS and thereby promote its externalization [55–58]. Moreover, Ebola VLPs lacking GP [18], which were generated by expressing GFP-fused VP40 alone, or using either vesicular stomatitis virus (VSV) [18] or Molony murine leukemia virus (MLV) pseudovirions [11] lacking GP, enter cells in a TIM-dependent manner with a similar efficiency [18], or more [11] efficiently than those bearing GP. These findings suggest a role for VP40 or viral protein(s) derived from pseudovirions in the externalization of PS on Ebola VLPs or pseudovirions, respectively. However, these studies also indicated that both VLPs and pseudovirions possessing Lassa virus (LASV) glycoprotein are internalized TIM-independently [11, 18], suggesting that PS exposure on virions is also dependent on the viral glycoproteins. To date, the impact of EBOV GP on the externalization of PS on Ebola VLPs has not been elucidated. Therefore, we generated VLPs lacking GP (VLPΔGP) by co-expressing VP40 and NP and examined whether PS was present on their surface. We conjugated individual VLPs contains equal amounts of protein to the beads. As expected, while Ebola VLP- (Fig 3A, middle) and VLPΔGP-conjugated beads (Fig 3A, right) were recognized by the anti-VP40 antibody with similar efficiency, the anti-GP antibody did not bind to the VLPΔGP-conjugated beads. We also found that binding of the anti-Xkr8 antibody to the VLPΔGP-conjugated beads was reduced by approximately 60% (Fig 3A, right and 3D, left). We further confirmed the incorporation of Xkr8 into the viral particles by using western blotting. Consistent with the results shown in Fig 3A, Xkr8 was incorporated efficiently into GP-positive viral particles, whereas Xkr8 was almost undetectable in VLPΔGP (Fig 3B). Moreover, we observed approximately one-third less binding of AF-ANX V to VLPΔGP-conjugated beads than to those bearing GP (Fig 3C and 3D, right). Together, these data indicate that GP mediates the trafficking of the host scramblase Xkr8 to the sites where VLPs bud and the subsequent incorporation of Xkr8 into VLPs, leading to exposure of PS on the surface of Ebola VLPs.

### A limited amount of PS is partly externalized in the fibrous structure in EBOV protein-expressing cells

It has previously been shown that VP40 alone can induce membrane curvature and scission from artificial membranes containing PS [57]. The same group also demonstrated that the association of VP40 with PS in the inner leaflet of the PM through its anionic charge leads to oligomerization of VP40 and subsequent extracellular exposure of PS, which as confirmed by showing that ANX V associates with the PM upon expression of VP40 alone [55, 56]. In contrast, another group demonstrated that EBOV infection does not induce apoptosis by showing limited binding of ANX V to EBOV-infected cells [59]. Here, we found that co-expression of VP40 and GP appeared to promote the externalization of PS on Ebola VLPs in a coordinated manner (Fig 3C), prompting us to further characterize the extracellular distribution of PS in cells expressing these EBOV proteins.

**Fig 4:**
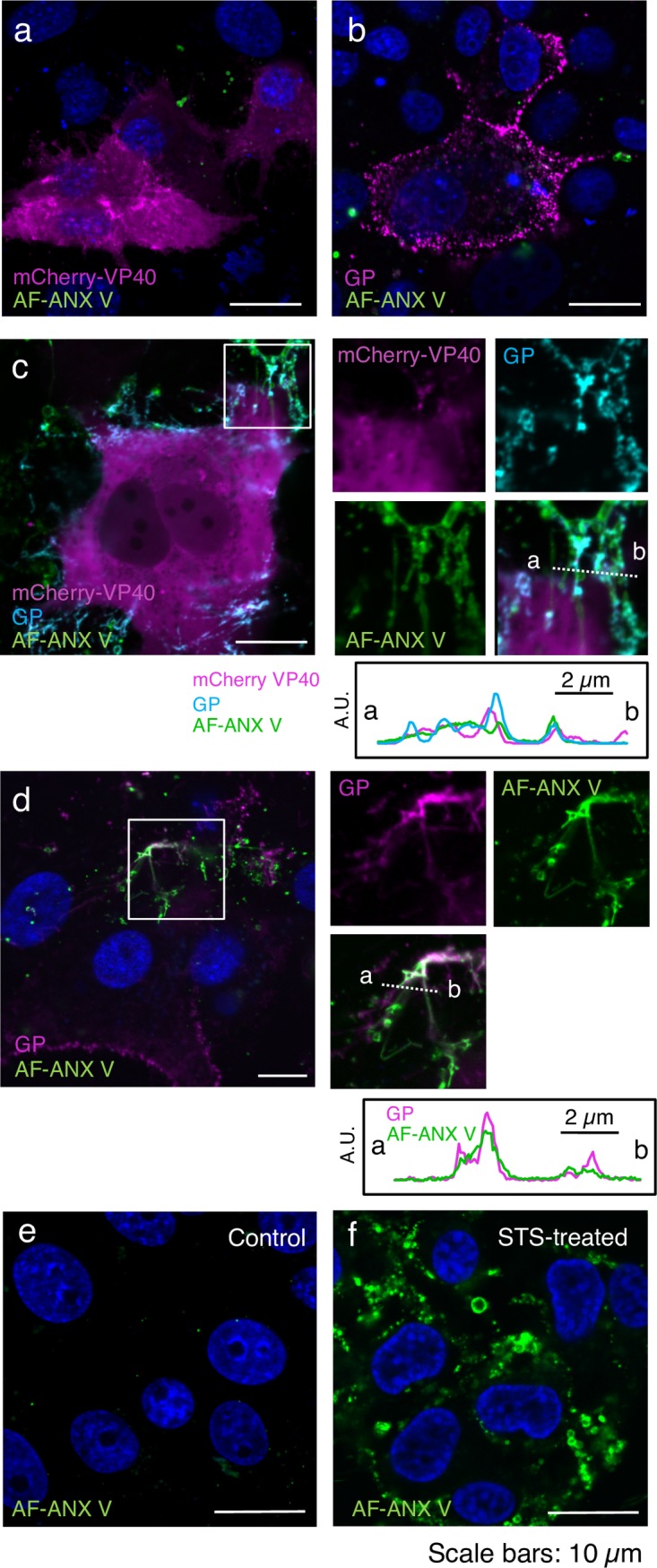
Distribution of extracellular PS in cells expressing EBOV proteins. Vero-E6 cells grown on 35-mm glass bottom dishes were transfected with the expression plasmids of mCherry-VP40 and wtVP40 at a ratio of 1:5 (a), GP alone (b), mCherry-VP40 and wtVP40 with GP (c), or wtVP40 and GP (d). At 48 h.p.t., the cells were harvested followed by AF-ANX V staining. For detection of GP, the cells were incubated in the medium containing the anti-GP antibody, followed by incubation with Alexa Fluor 647-conjugated secondary antibody. After being washed with medium and ANX V binging buffer, the cells were treated with AF-ANX V. After washing again, the AF-ANX V signal (green) and EBOV proteins were observed by using a confocal microscope. Individual viral proteins are shown in magenta (a, b, and d). In panel (c), mCherry-VP40 and GP are shown in magenta and cyan, respectively. As a control, a backbone plasmid was transfected (e). Panel (f) represents Vero-E6 cells treated with 1 μM STS for 6 h. The nuclei (blue) were counterstained with Hoechst 33342. Insets show the boxed areas. The plots indicate the individual fluorescence intensity along each of the corresponding lines. A.U.; arbitrary unit. Scale bars in the large panels: 10 μm.

We assessed the extracellular distribution of PS by using AF-ANX V upon expression of VP40 with or without GP in Vero-E6 cells. To preserve the intact intracellular distribution of the phospholipids, AF-ANX V labeling was carried out in live cells. AF-ANX V successfully detected extracellular PS on the surface of STS-induced cells, which was often associated with extensive blebs that are a common feature of apoptosis [60] (Fig 4F, green). We visualized VP40 in live cells by co-expressing mCherry-VP40 and wtVP40 at a ratio of 1:5, which led to the accumulation of mCherry signals in the PM (Fig 4A, magenta). GP, which was visualized with the anti-GP antibody, was distributed in an intense, specked pattern on the surface of the cells (Fig 4B, magenta). At 48 h post-transfection (h.p.t.), limited-to-no AF-ANX V signal was detected on the surface of mCherry-VP40- and GP-positive cells (Fig 4A and 4B, green). We obtained similar results with cells at 72 h.p.t. (S5 Fig). We also co-expressed mCherry-VP40 and wtVP40 (1:5) with GP and confirmed that GP (Fig 4C, cyan) often localized in mCherry-positive filamentous structures (Fig 4C, magenta). We observed that AF-ANX V partly associated with the mCherry- and GP-positive filamentous architectures (Fig 4C, green), although the frequency of the presence of AF-ANX V remained low. To exclude the possibility that fusion of mCherry to VP40 was driving inappropriate oligomerization and a subsequent defect in externalization of PS, we co-expressed wtVP40 and GP, and then stained the cells with the anti-GP antibody (Fig 4D, magenta). We identified the cells expressing both VP40 and GP in terms of their distinct filamentous distribution of GP (Fig 4B, magenta vs. Fig 4C, cyan and Fig 4D, magenta). Consistent with the result shown in Fig 4C, only a limited amount of AF-ANX co-localized with the filamentous GP signals (Fig 4D, green). These data suggest that externalization of PS appears to be restricted to the filamentous structures upon co-expression of VP40 and GP, and in the released VLPs (Figs 1C, 3C and 3D).

### Incorporated Xkr8 contributes to the externalization of PS on Ebola VLPs

**Fig 5:**
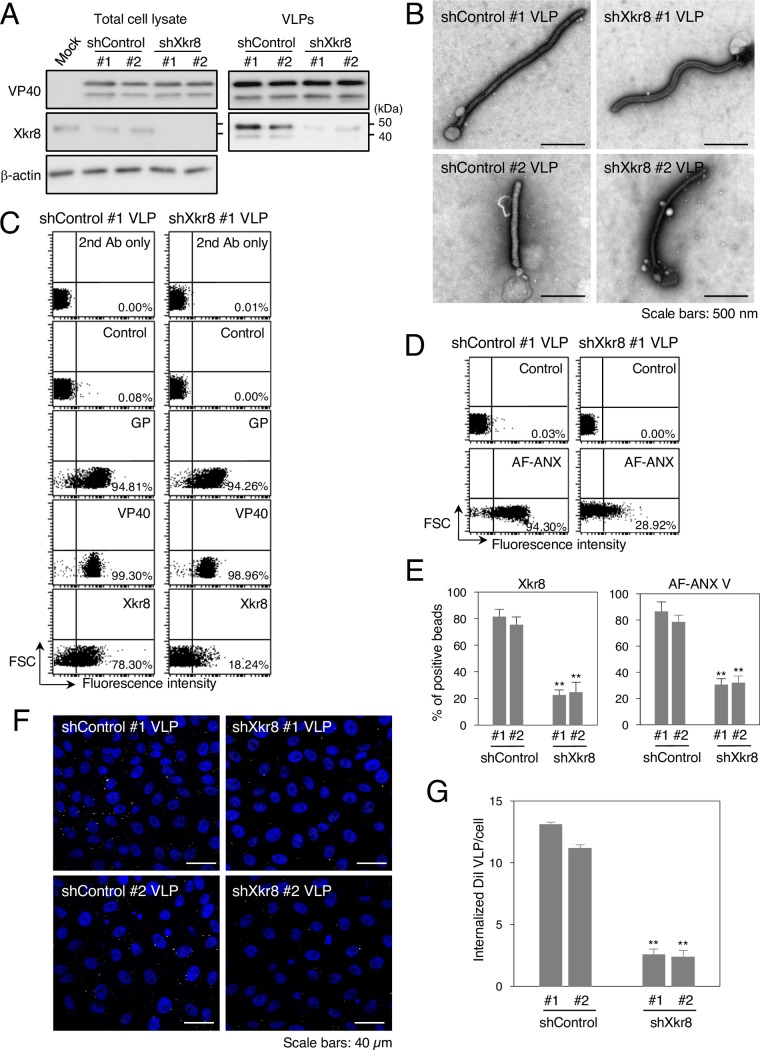
Role of Xkr8 in the externalization of PS on Ebola VLPs and PS-dependent VLP internalization. (A) Downregulation of Xkr8 by shRNA. Individual 293T clones transduced by shRNA plasmids were transfected with the expression plasmids of EBOV VP40, GP, and NP. At 48 h.p.t., the cells and culture medium were harvested. Viral particles were purified from culture medium by ultracentrifugation. Expression of VP40 and Xkr8 in total cell lysates and VLPs was analyzed by western blotting with rabbit polyclonal antibodies against VP40, Xkr8, or β-actin. (B) Negative staining of VLPs obtained from individual shRNA clones. Individual shRNA cell clones were transfected with the expression plasmids of VP40, GP, and NP. At 48 h.p.t., the culture medium was harvested. Viral particles were purified from the culture medium by ultracentrifugation followed by negative staining. Scale bars; 500 nm. (C–E) The effect of Xkr8 knockdown on the incorporation of viral proteins and Xkr8 into VLPs and on the externalization of PS on the surface of Ebola VLPs. (C) Ebola VLPs obtained from shControl #1 (left) or shXkr8 #1 (right) clones were conjugated with latex beads. The beads were then incubated with rabbit polyclonal antibodies against EBOV GP, VP40, or Xkr8 followed by flow cytometric analysis. 2^nd^ Ab indicates samples that were not treated with primary antibody. As a control, the rabbit anti-LASV GPC polyclonal antibody was used. (D) For detection of externalized PS on the VLPs, the beads were incubated with AF-ANX V and subsequently subjected to flow cytometric analysis. The percentages of the positive populations are indicated. X-axis: fluorescence intensity, Y-axis: forward scatter corner signals. The results are representative of three individual experiments. (E) Summary of the binding of the anti-Xkr8 antibody (left) and AF ANX V (right) to individual shControl VLP- or shXkr8 VLP-conjugated beads. Each experiment was performed in triplicate and the percentages of the positive populations are presented as the mean ± SD. **, *P* < 0.01 versus respective control (Student’s *t* test). (F, G) The effect of Xkr8 knockdown on the internalization of VLPs. (F) Purified VLPs from individual shRNA clones were labeled with DiI and adsorbed to Vero-E6 cells for 30 min at room temperature. After incubation for 2 h at 37°C, surface-bound virions were removed by trypsinization and the internalization of Ebola VLPs was analyzed by using a confocal laser scanning microscope. (G) The number of internalized DiI-VLPs in each of 10 individual clones was measured. Each experiment was performed in triplicate and the relative uptake efficiencies are presented as the mean ± SD. **, *P* < 0.01 versus respective control (Student’s *t* test).

We next examined the role of the incorporated Xkr8 in the externalization of PS on the surface of Ebola VLPs. It has been shown that downregulation of Xkr8 prevents PS exposure in the PM of apoptotic cells and their subsequent uptake by phagocytes [27]. We downregulated the expression of Xkr8 in VLP-producing HEK293T cells by using short hairpin RNA (shRNA). We confirmed the expression of VP40 in total cell lysates and VLPs by western blotting, in which VP40 was observed as a doublet as previously reported [40]. The efficiency of Xkr8 knockdown in two cell clones was confirmed by western blotting (Fig 5A, left). The results of western blotting (Fig 5A, right) and negative staining (Fig 5B), confirmed that downregulation of Xkr8 affected neither the production nor the morphology of the VLPs. We further assessed the incorporation of VP40, GP, and Xkr8 in viral particles by using a latex bead-based analysis. VP40 and GP were incorporated into VLPs produced in Xkr8 knockdown cells (shXkr8 VLPs) similarly to those derived from control clones. In contrast, incorporation of Xkr8 decreased by approximately 70%–75% in shXkr8 VLPs (Fig 5C and 5E), which is consistent with the western blot results. We also examined the effect of Xkr8 knockdown on the externalization of PS in individual VLPs. Remarkably, AF-ANX V binding to shXkr8 VLPs decreased to about 60%–65% of that of the control Ebola VLPs (Fig 5D and 5E).

### Xkr8-mediated externalization of PS on Ebola VLPs is important for uptake by target cells

Because Xkr8 appeared to mediate exposure of PS on the surface of Ebola VLPs (Fig 3), we next assessed the impact of Xkr8 on the internalization of VLPs. We previously established a live-cell imaging system to evaluate VLP internalization [4]. Purified VLPs was fluorescently labeled with a lipophilic tracer DiI, which is incorporated into the envelope of the virions. DiI-VLPs were adsorbed to Vero-E6 cells by incubating for 30 min at room temperature. After washing to remove unbound VLPs, the cells were incubated for 2 h at 37°C. Cell surface-bound, uninternalized VLPs were then removed by trypsinization and intracellular DiI-signals were analyzed by using a confocal laser scanning microscope. As shown in Fig 5F and 5G, shXkr8 VLPs were internalized significantly less efficiently than were control VLPs. These results demonstrate that Xkr8 incorporated in VLPs promotes the scrambling of PS in the envelope, leading to their PS-dependent uptake.

### A pan-caspase inhibitor blocks Xkr8-mediated externalization of PS on Ebola VLPs and their subsequent internalization

**Fig 6:**
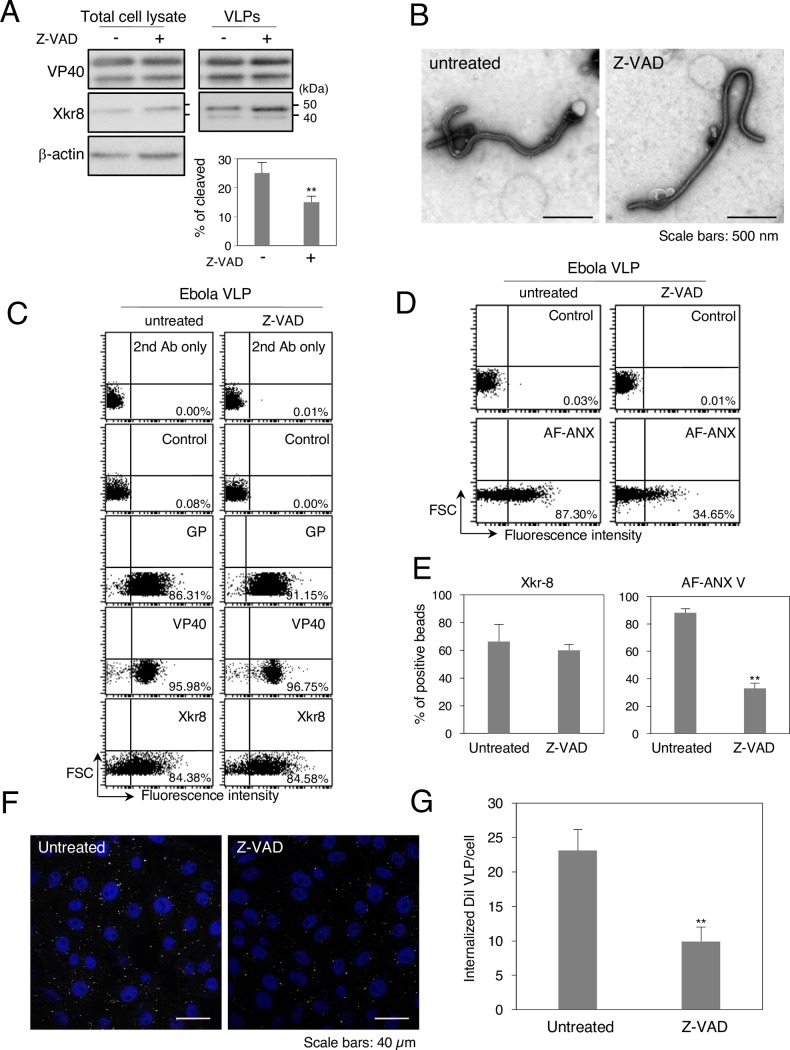
Effect of a pan-caspase inhibitor on the externalization of PS on the surface of Ebola VLPs and their internalization. (A) The effect of a pan-caspase inhibitor on Xkr8 in cell lysate and VLPs. 293T cells were transfected with the expression plasmids of EBOV VP40, GP, and NP and incubated for 48 h in the absence or presence of 20 μM Z-VAD-FMK. At 48 h.p.t., the cells and culture medium were harvested. Viral particles were purified from culture medium. Total cell lysates and VLPs were analyzed by western blotting with rabbit polyclonal antibodies against VP40, Xkr8, or β-actin. % of cleaved Xkr8 was analyzed as the ratio of the intensities of the cleaved bands to total Xkr8 bands. (B) The effect of Z-VAD-FMK treatment on the morphology of VLPs. 293T cells were transfected with the expression plasmids of EBOV VP40, NP, and GP and incubated for 48 h in the absence or presence of 20 μM Z-VAD-FMK. At 48 h.p.t., the culture medium was harvested. Viral particles were purified from the culture medium by ultracentrifugation followed by negative staining. Scale bars; 500 nm. (C–E) The effect of Z-VAD-FMK treatment on the incorporation of viral proteins and Xkr8 into VLPs and on the externalization of PS on the surface of Ebola VLPs. (C) Ebola VLPs obtained from untreated- (left) or Z-VAD-FMK-treated cells (right) were conjugated with latex beads. The beads were then incubated with rabbit polyclonal antibodies against EBOV GP, VP40, or Xkr8 followed by flow cytometric analysis. 2^nd^ Ab indicates samples that were not treated with primary antibody. As a control, the rabbit anti-LASV GPC polyclonal antibody was used. (D) For detection of externalized PS on the VLPs, the beads were incubated with AF-ANX V and subsequently subjected to flow cytometric analysis. The percentages of the positive populations are indicated. X-axis: fluorescence intensity, Y-axis: forward scatter corner signals. The results are representative of three individual experiments. (E) Summary of the binding of the anti-Xkr8 antibody (left) and AF ANX V (right) to VLPs released from untreated- or Z-VAD-FMK-treated cells. Each experiment was performed in triplicate and the percentages of the positive populations are presented as the mean ± SD. **, *P* < 0.01 versus respective control (Student’s *t* test). (F, G) The effect of Z-VAD-FMK treatment on VLP internalization. (F) Purified VLPs were labeled with DiI and adsorbed to Vero-E6 cells for 30 min at room temperature. After incubation for 2 h at 37°C, surface-bound virions were removed by trypsinization for 5 min at 37°C and the internalization of the Ebola VLPs was analyzed by using a confocal laser scanning microscope. (G) The number of internalized DiI-VLPs in 10 individual cells was measured. Each experiment was performed in triplicate and the relative uptake efficiencies are presented as the mean ± SD. **, *P* < 0.01 versus respective control (Student’s *t* test).

Xkr8 possesses a recognition sequence for caspases 3 and 7 in its C-terminus and is converted into an active form by caspases upon apoptotic stimuli [27, 28]. Yet, one study demonstrated that EBOV infection does not induce apoptosis by showing limited binding of ANX V to EBOV-infected cells, although limited activation of caspase was also observed in the infected cells [45, 59]. We therefore tested whether Xkr8 is activated upon co-expression of VP40 and GP. Western blot analysis revealed Xkr8 as single band (45 kDa) in the cell lysate, but upon co-expression of viral proteins, Xkr8 incorporated in VLPs was detected as a doublet (Figs 3B and 5A). The predicted size of the cleaved protein is approximately 39 kDa, which is identical to the size of the lower band. We further demonstrated that the intensity of the lower Xkr8 band decreased following treatment with the pan-caspase inhibitor Z-VAD-FMK (Fig 6A), suggesting that the limited caspase activation upon expression of viral proteins may trigger the activation of Xkr8 incorporated in VLPs. We confirmed that the inhibitor treatment had no effect on the production or morphology of the VLPs (Fig 6A and 6B). While Z-VAD-FMK treatment did not affect the incorporation of viral proteins or Xkr8 into the viral particles (Fig 6C and 6E), it led to the significant suppression of the binding of AF-ANX V to the VLP-conjugate beads (Fig 6D and 6E), suggesting that exposure of PS on VLPs is dependent on caspase-mediated activation of Xkr8. Finally, we asked whether the PS-dependent uptake of Ebola VLPs was also dependent on the caspase-mediated activation of Xkr8 in virions. As shown in Fig 6F and 6G, the uptake of VLPs released from the cells treated with Z-VAD-FMK was downregulated by about one-third compared with that of VLPs released from untreated cells. These data indicate that Xkr8 incorporated in Ebola VLPs is cleaved in a caspase-dependent manner, which mediates the exposure of PS on the viral particles and promotes their subsequent internalization.

## Discussion

Accumulating evidence indicates that a variety of enveloped viruses exploit apoptotic mimicry for their efficient entry [61]. However, the mechanism underlying the externalization of PS on the surface of virions is poorly understood. Because the distribution and topology of PS vary among the intracellular organelles, the mechanisms by which viruses acquire a PS-rich envelope rely on their assembly strategies. Since the luminal leaflet of the ER is enriched with PS, budding into the ER is one potential mechanism by which viruses acquire an envelope with externalized PS. In fact, the flaviviruses, which enter cells via a PS-dependent mechanism, bud their progeny virions into the ER [62]. Vaccinia virus derives its viral envelope from the ER and membrane sheets that are generated upon rupture of the ER cisternae [55, 63]. Poliovirus infection generates autophagosome-like double-membraned organelles enriched with PS in both the luminal and cytoplasmic leaflets. This specific organelle originates from the ER and is ER-oriented, allowing clusters of viral particles to be encapsulated, non-lytically released, and efficiently enter target cells [10]. Several envelop viruses that exploit apoptotic mimicry bud from the PM, in which most of the PS is usually distributed on the inner leaflet of the membrane. Since infection by various viruses induces apoptosis in their target cells [64–69], it is likely that these viruses bud from the PM of apoptotic cells to acquire viral envelopes that contain PS in their outer leaflets.

**Fig 7:**
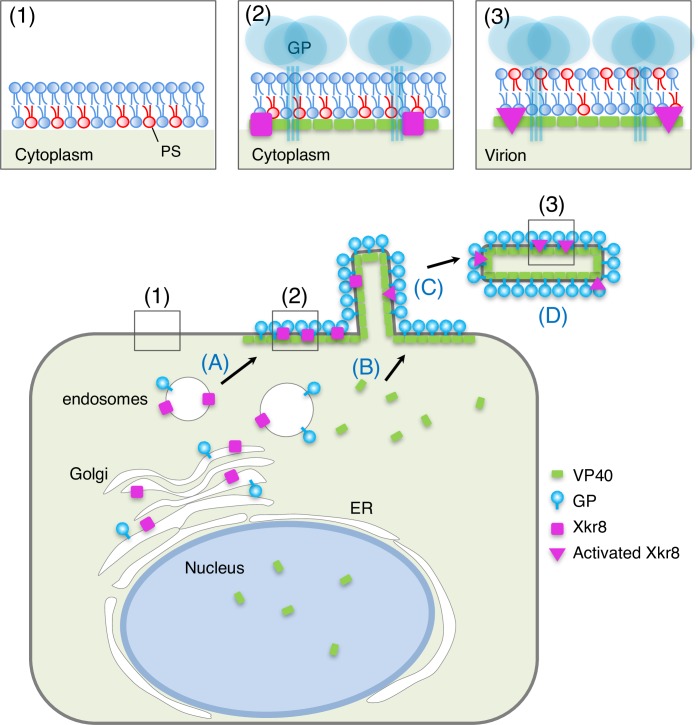
Role of Xkr8 in PS exposure on the surface of virus particles. Xkr8 is transported to the budding sites together with GP *via* intracellular vesicles (A). VP40 is independently transported to the PM (B). Transported GP and Xkr8 are incorporated into virus particles (C). Incorporated Xkr8 is activated by a caspase (D), which leads to externalization of PS in the envelope of the EBOV particles (1–3).

However, it has been shown that EBOV infection is not associated with the induction of apoptosis [59]. In the present study, we identified the scramblase Xkr8 as a factor for the exposure of PS on the surface of Ebola VLPs (Fig 5). As illustrated in Fig 7, expression of VP40 together with GP promotes the intracellular, vesicle-mediated trafficking of Xkr8 to the PM where both viral proteins accumulate (Fig 2), and the subsequent incorporation of Xkr8 into VLPs (Fig 3), and PS externalization (Figs 1 and 3). We further found that Xkr8 exhibits its scramblase activity in a caspase-dependent manner (Fig 6). Therefore, Ebola virus may be able to exploit PS-mediated internalization without causing apoptosis.

We observed that AF-ANX V partly associates with filamentous structures in the cells expressing VP40 and GP (Fig 4), which suggests that the asymmetrical distribution of PS in the PM is retained until VLP formation (Figs 1C and 3C). Such highly restricted PS exposure appears to be important for the infected cells to escape from phagocytosis.

The mechanism underlying the externalization of PS remains to be elucidated. Western blot analyses indicated that VLPs contained both intact and cleaved forms of Xkr8, whereas only full-length Xkr8 was detected in the cell lysates (Figs 3, 5 and 6). Moreover, treatment with a pan-caspase inhibitor suppressed both PS externalization on the VLPs and their internalization (Fig 6). These results suggest that PS exposure is regulated by the restricted activation of Xkr8 in VLPs. It is likely that activation of Xkr8 is mediated by the limited activation of caspase that was observed in EBOV-infected cells [59], although the mechanism underlying caspase activation in EBOV-infected cells remained to be clarified.

The GP-dependent Xkr8-induced PS exposure on viral particles that we uncovered in this study may be shared by other viruses, since the glycoproteins of viruses that bud from the PM are likely trafficked via the same pathway for post-translational modification. A previous study showed that expression of Xkr8 is epigenetically regulated in certain cell types [27], suggesting that its role in viral entry might rely on the tropism of viruses.

Ebola VLPs [18], and VSV- or MLV-based pseudovirions [11] that lack GP have been shown to enter the cells in a TIM-dependent manner with similar [18], or greater [11] efficiency than those bearing GP. However, we previously demonstrated that the efficiencies of adsorption to the cell surface and subsequent internalization of VLPs that lacked GP are low, supporting a requirement for GP in EBOV entry [11]. Consistently, in our present study, we observed that VLPΔGP possessed less PS exposure on the surface than did intact Ebola VLPs (Fig 3C and 3D). This discrepancy might arise from differences in the experimental systems. We generated VLPΔGP by co-expressing VP40 and NP, rather than preparing VLPs by expressing GFP-fused VP40 alone [18], which might yield viral particles with different morphological features. Moreover, in a pseudovirus system, the matrix proteins originate from the backbone viruses, suggesting that the distribution of PS in the viral envelopes may not reflect that of authentic EBOV virions.

Suzuki *et al*. have evaluated the expression level of mouse Xkr8 in various tissues [27]. Investigation of Xkr8 expression in the cell types that EBOV targets may corroborate the significance of Xkr8-dependent externalization of PS on EBOV particles.

In summary, in addition to its established functions in the viral entry process, our findings support a role for GP in affecting the topology of PS in viral particles, which is mediated by recruiting a scramblase to the viral particles. We demonstrated that a blocking antibody against TIM-1 efficiently blocked the internalization of EBOV particles [13]. Since apoptotic mimicry is an essential process for entry into host cells for many types of viruses, this pathway represents a potential target for new antiviral drug design.

## Methods

### Cell culture and transfection

African green monkey kidney epithelial Vero-E6 cells and human embryonic kidney HEK293T cells (American Type Culture Collection) were grown in high-glucose Dulbecco’s modified Eagle’s medium (DMEM) containing 10% fetal bovine serum (FBS) and antibiotics. HEK293T cell lines transduced by pLKO.1 lentiviral vectors were maintained in the presence of 1 μg/ml puromycin. Cells were maintained at 37°C in 5% CO_2_. Transfection of Vero-E6 and HEK293T cells was carried out with TransIT-LT1 (Mirus, Madison, WI).

### Plasmids and reagents

An expression plasmid of EBOV VP40 fused to mCherry (mCherry-VP40) was constructed by insertion of VP40 cDNA into pmCherry-C1 plasmids (Addgene, Cambridge, USA) at the EcoRI and BamHI sites. VP40 cDNA was amplified by PCR. Oligonucleotides used for PCR are as follows: Forward 5’-GGAATTCTATGAGGCGGGTTATA-3’; Reverse 5’- CGGGATCCTTACTTCTCAATCACAGC-3’. pLKO.1 plasmids encoding a short hairpin RNA sequence targeting human Xkr8 were purchased from GE healthcare Dharmacon Inc. (Lafayette, USA). 1,1'-dioctadecyl-3,3,3',3'-tetramethylindocarbocyanine perchlorate (DiI), and Alexa Fluor 488-labeled Annexin V (AF-ANX V) were purchased from Thermo Fisher Scientific (Waltham, USA). Unlabeled ANX V was purchased from Abcam (Cambridge, UK). Staurosporin (STS) was purchased from Wako pure chemical industries Ltd. (Osaka, Japan). N-Benzyloxycarbonyl-Val-Ala-Asp(O-Me) fluoromethyl ketone (Z-VAD-FMK) was purchased from Merck (Darmstadt, Germany).

### Purification of Ebola VLPs

Purification of Ebola VLPs has been described previously [4]. Briefly, equal amounts of the pCAGGS expression plasmids for EBOV subtype Zaire, strain Mayinga VP40 [41], GP [49], and NP [37] were transfected into HEK293T cells. For Z-VAD-FMK treatment, 20 μM Z-VAD was added to the medium after transfection. Forty-eight hours post-transfection (h.p.t.), the culture supernatants were harvested, and centrifuged at 1,500 rpm for 5 min and then at 3,500 rpm for 15 min to remove detached cells and cell debris, respectively. The VLPs were precipitated through a 30% sucrose cushion by centrifugation at 11,000 rpm for 1 h at 4°C with an SW28 rotor (Beckman, Fullerton, USA). Precipitated VLPs were resuspended in TNE buffer [10 mM Tris-HCl (pH 7.6), 100 mM NaCl, 1 mM EDTA], and fractionated by use of a 20%–60% sucrose gradient in TNE buffer at 27,000 rpm for 2.5 h at 4°C with an SW40 rotor (Beckman). Incorporation of VP40, GP, and Xkr8 in the purified VLPs was confirmed by western blot analysis with a mouse monoclonal antibody against VP40 (clone 6; 1:4000 dilution), GP (clone 133.13.16; 1:4000 dilution), and a rabbit anti-Xkr8 polyclonal antibody (Abcam; 1:250 dilution). Morphology of VLPs was confirmed by negative staining. Protein concentrations of VLPs were measured by use of a Bradford protein assay kit (BioRad, Hercules, USA).

### Preparation of liposomes

For preparation of liposomes consisting of PC alone (liposome PC), 125 μl of 1 mM egg yolk phosphatidylcholine (EPC; NOF corporation, Tokyo, Japan) was dissolved in 125 μl chloroform. The solution was dried in a vacuum evaporator and the lipid film was resuspended in 250 μl 10 mM HEPES buffer (pH 7.4). After incubation for 10 min at room temperature, the solution was sonicated for 15–30 sec at room temperature in the water bath. The size and apparent zeta potential of liposomes were analyzed with Zetasizer Nano ZS (ZEN3600, Malvern Instruments Ltd., Malvern, UK). For preparation of liposomes consisting of PC and PS in the ratio of 1:3 (liposome PC/PS), 31.25 μl 1 mM EPC and 93.75 μl 1 mM L-α-PS (Sigma-Aldrich, St. Louis, USA) were dissolved in 125 μl chloroform, followed by the same procedure as described above.

### Characterization of the surface molecules of Ebola VLPs

The surface molecules of Ebola VLPs were characterized by using a flow cytometry-based analysis [33, 34]. Ten micrograms of purified Ebola VLPs or FBS, or 10 mM of liposomes were incubated with 10 μl of 4-μm aldehyde/surface latex beads (Thermo Fisher Scientific) for 15 min at room temperature in 100 μl of PBS containing 1% BSA, followed by a 2-h incubation with gentle shaking in 1 ml of PBS containing 1% BSA at room temperature. The reaction was stopped by incubation for 30 min at room temperature in the presence of 100 mM glycine. Conjugated beads were washed three times in 1% BSA in PBS and incubated with a rabbit polyclonal antibody against GP (1:1000 dilution), VP40 (1:1000 dilution), or Xkr8 (Rockland Immunochemicals Inc., Limerick, USA; 1:200 dilution) for 1 h at room temperature, followed by incubation with Alexa Fluor 488-conjugated secondary antibodies (Thermo Fisher Scientific; 1:1000 dilution). As a control, a rabbit anti-LASV GPC polyclonal antibody (1:1000 dilution) was used. For detection of VP40 and Xkr8 in VLPs, VLPs were permeabilized by incubating them in the presence of 0.05% Triton X-100 in PBS for 10 min at room temperature. For detection of PS, the beads were washed three times in 1% BSA in PBS, and once in ANX V binding buffer [20 mM Hepes (pH 7.4), 140 mM NaCl, 2.5 mM CaCl_2_], followed by incubation with 1.2 μg/ml Alexa Fluor 488-labeled ANX V. For blocking analysis, the beads were pre-incubated in the presence of various concentrations of unlabeled ANX V for 2 h at room temperature in ANX V binding buffer. After being washed in the same buffer, the beads were incubated with AF-ANX V as described above. After another wash in the same buffer, antibody or AF-ANX V binding to the beads was analyzed by using FACSCalibur (Becton Dickinson, San Diego, USA).

### Immunofluorescence staining

Vero-E6 cells grown on coverslips were transfected with the expression plasmids of VP40, mCherry-VP40, GP, or NP. At 48 h.p.t., the cells were fixed with 4% paraformaldehyde (PFA) in PBS for 10 min at room temperature, permeabilized with PBS containing 0.05% Triton X-100 for 10 min at room temperature, and blocked in PBS containing 4% BSA for 20 min at room temperature The cells were then incubated with a mouse monoclonal antibody against VP40 (clone 6; 1:2000 dilution), GP (clone 133.13.16; 1:2000 dilution), NP (clone 7.42.18; 1:2000 dilution), or a rabbit polyclonal antibody against Xkr8 (1:200 dilution) for 1 h at room temperature For co-immunofluorescence staining of VP40 and GP, a mouse monoclonal antibody against VP40 and a rabbit polyclonal antibody against GP (1:2000 dilution) were used. The cells were then washed three times in PBS and incubated with Alexa Fluor 488, 594, or 647-labeled secondary antibodies (1:2000 dilution) (Thermo Fisher Scientific) for 1 h at room temperature After washing, nuclei were counterstained with Hoechst 33342 (Cell Signaling Technology, Trask Lane, USA). Images were collected with the 60 × oil-immersion objective lens of a confocal laser scanning microscope (Fluoview FV10i, Olympus, Tokyo, Japan) and acquired by using FV10-ASW software (Olympus). Line scan imaging was performed by using FV10-ASW software.

### Characterization of the extracellular distribution of PS

For analysis of the extracellular distribution of PS, Vero-E6 cells grown on 35-mm glass bottom dishes (MatTek corporation, Ashland, USA) were transfected with the expression plasmids of mCherry-VP40 and wtVP40 at a ratio of 1:5, and/or GP. At 48 h.p.t., the cells were incubated with a rabbit polyclonal antibody against GP (1:2000 dilution) for 1 h at 37°C. After washing in the medium, the cells were incubated with Alexa Fluor 647-conjugated secondary antibody (1:1000 dilution) for 30 min at 37°C. After again washing the cells in the medium, the medium was replaced with ANX V binding buffer and the cells were incubated in the presence of 1.2 μg/mL AF-ANX V in the same buffer for 15 min at room temperature. The fluorescent signals of ANX V along with viral proteins were analyzed by use of a confocal laser scanning microscope. The nuclei were counterstained with Hoechst 33342.

### Knockdown of Xkr8 expression by shRNA

For downregulation of Xkr8 in HEK293T cells, the cells were transduced with an HIV-based lentiviral pLKO.1 vector of a shRNA encoding the corresponding target sequence 5’-AACTTCTGTGCTAAATGGGTC-3’ (GE healthcare Dharmacon Inc., Lafayette, USA). As a control, a pLKO.1 plasmid encoding a sequence that does not target any known genes (Sigma-Aldrich) was used. Recombinant lentiviruses were generated by co-transfection of the pLKO.1 vector, pCAGGS-HIV gag/pol, pCAGGS-Rev, and pCMV-VSV-G in HEK293T cells. The culture medium was collected at 48 and 72 h.p.t., and concentrated 100-fold by ultracentrifugation at 38,000 rpm for 2 h at 4°C with an 80Ti rotor (Beckman). For lentiviral infections, HEK293T cells (1 x 10^5^) were grown in 24-well plates, the culture medium was replaced with ice-cold DMEM supplemented with 10% FBS and 20 mM HEPES (pH 7.4), and the cells were incubated with viral stocks for 1 h at 4°C at a multiplicity of infection (m.o.i) of 5. After being washed twice with complete medium, the cells were cultured in complete medium for 48 h, and were then cultured under selection in the presence of 1 μg/ml puromycin. Single puromycin-resistant colonies were picked up and Xkr8 expression was confirmed by western blotting.

### Electron microscopy

Transmission electron microscopy (TEM) was carried out as described previously [70, 71]. Ebola VLP-conjugated beads fixed with 2.5% glutaraldehyde were adsorbed to glow discharged 400-mesh formvar-coated copper mesh grids and then negatively stained with 1% uranyl acetate. Purified VLPs fixed with 0.25% glutaraldehyde were adsorbed to collodion-carbon-coated copper grids and negatively stained with 2% phosphotungstic acid solution (pH 5.8). Ebola VLP-conjugated beads or purified VLPs were examined with a Tecnai F20 electron microscope (FEI) at 200 kV or an H-7650 electron microscope (Hitachi) at 80 kV, respectively.

### Internalization of Ebola VLPs

Fluorescent labeling and an internalization assay of Ebola VLPs have been described previously [4]. For fluorescent labeling of VLPs, 1 ml of fractionated VLPs (100 ng/ml) was incubated with 6 μl of 10 μM stock solution of DiI in the dark for 1 h at room temperature with gentle agitation. For analysis of the internalization of DiI-labeled EBOV VLPs, Vero-E6 cells were grown in 35-mm glass-bottom culture dishes, washed in 1 ml of phenol red-free MEM (Thermo Fisher Scientific) containing 2% FBS and 4% BSA, and incubated with DiI-labeled Ebola VLPs in 50 μl of the same medium at room temperature for 30 min. The cells were then washed with the medium and incubated for 1 h at 37°C. Surface-bound VLPs were removed by trypsin and the internalization of the DiI-VLPs was analyzed by use of a confocal laser scanning microscope (Fluoview FV10i). For the internalization analysis, five fields were acquired randomly, and the number of DiI-labeled virions was measured in approximately 250 individual cells in an automated fashion by using MetaMorph software (Molecular Devices, Sunnyvale, USA). Each experiment was performed in triplicate and the results are presented as the mean ± standard deviation.

## Supporting information

S1 Fig Protease protection assay. Ebola VLP-conjugated beads were treated with or without 0.1 mg/ml trypsin in the presence or absence of 0.05% Triton X-100 at room temperature for 30 min, followed by the addition of 5 mg/ml soybean trypsin inhibitor. After being washed in PBS containing 2% BSA, the beads were incubated with rabbit polyclonal antibodies against VP40 followed by incubation with Alexa Fluor488-labeled secondary antibody. The binding of antibody to the beads was analyzed by flow cytometry. The percentages of the positive populations are indicated. 2^nd^ Ab represents the beads that were not treated with primary antibody. X-axis: fluorescence intensity, Y-axis: forward scatter corner signals. The results are representative of three individual experiments.
(TIFF)

S2 Fig Effect of Triton X-100 on the binding of antibodies to the Ebola VLP-conjugated beads. Ebola VLP-conjugated beads were incubated with or without 0.05% Triton X-100 in PBS containing 2% BSA for 10 min at room temperature. After being washed, the beads were incubated with rabbit polyclonal antibodies against EBOV GP, VP40, or LASV GPC, followed by incubation with Alexa Fluor 488-labeled secondary antibody. The binding of antibody to the beads was analyzed by flow cytometry. The percentages of the positive populations are indicated. 2^nd^ Ab represents the beads that were not treated with primary antibody. X-axis: fluorescence intensity, Y-axis: forward scatter corner signals. The results are representative of three individual experiments.
(TIFF)

S3 Fig Intracellular distribution of endogenous and exogenously expressed Xkr8 in human cells. HEK293T cells (a), HEK293T cells transiently expressing FLAG- (b) or GFP-tagged Xkr8 (c), and NU-GC-3 cells (d) grown on cover slips were fixed in 4% PFA followed by immunofluorescent staining with the rabbit polyclonal anti-Xkr8 antibody (a and d), or rabbit polyclonal anti-FLAG antibody (b) (Cell Signaling Technology). The intracellular distribution of endogenous or tagged Xkr8 was analyzed by using a confocal laser scanning microscope. The nuclei (blue) were counterstained with Hoechst 33342. Scale bars, 10 μm.
(PDF)

S4 Fig Xkr8 and GP localize together in Rab7-positive endosomes. Vero-E6 cells stably expressing eGFP-Rab7 [4, 72] were transfected with an expression plasmid of EBOV GP. At 48 h.p.t., cells were fixed in 4% PFA and subjected to immunofluorescence staining with a rabbit anti-Xkr8 and anti-GP polyclonal antibodies. Insets show the boxed areas. eGFP-Rab7, GP, and Xkr8 are shown in green, cyan, and magenta, respectively. A and B represent boxed areas in the image. The plot indicates the relative fluorescence intensity of the individual channels along each of the corresponding lines. A.U.; arbitrary unit. Scale bar: 10 μm.
(TIFF)

S5 Fig Distribution of extracellular PS in cells expressing EBOV proteins. Vero-E6 cells grown on 35-mm glass bottom dishes were transfected with the expression plasmids of mCherry-VP40 and wtVP40 at a ratio of 1:5 (a), GP alone (b). At 72 h.p.t., the cells were harvested and followed by AF-ANX V staining. For detection of GP, the cells were incubated in the medium containing the anti-GP antibody, followed by incubation with Alexa Fluor 647-conjugated secondary antibody. After being washed with medium and ANX V binging buffer, the cells were treated with AF-ANX V. After washing again, the AF-ANX V signal (green) and EBOV proteins (magenta) were observed by using a confocal microscope. The nuclei (blue) were counterstained with Hoechst 33342. Scale bars : 10 μm.
(TIFF)
