# Supplementary figures and images for: Ebola virus requires a host scramblase for externalization of phosphatidylserine on the surface of viral particles
Source: PLoS Pathog. 2018 Jan 16;14(1):e1006848. doi: 10.1371/journal.ppat.1006848 (PMC5786336; doi:10.1371/journal.ppat.1006848)

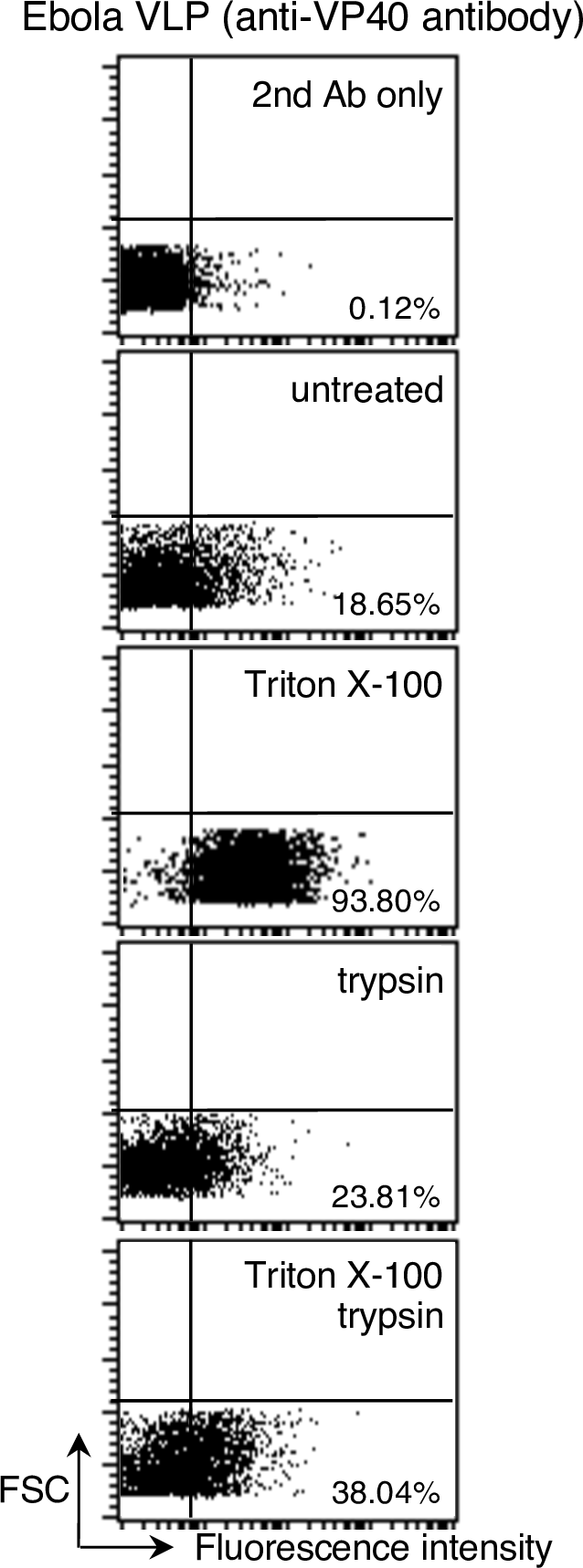

Supplement: S1 Fig — Ebola VLP-conjugated beads were treated with or without 0.1 mg/ml trypsin in the presence or absence of 0.05% Triton X-100 at room temperature for 30 min, followed by the addition of 5 mg/ml soybean trypsin inhibitor. After being washed in PBS containing 2% BSA, the beads were incubated with rabbit polyclonal antibodies against VP40 followed by incubation with Alexa Fluor488-labeled secondary antibody. The binding of antibody to the beads was analyzed by flow cytometry. The percentages of the positive populations are indicated. 2nd Ab represents the beads that were not treated with primary antibody. X-axis: fluorescence intensity, Y-axis: forward scatter corner signals. The results are representative of three individual experiments. (TIFF) [file ppat.1006848.s001.tiff]

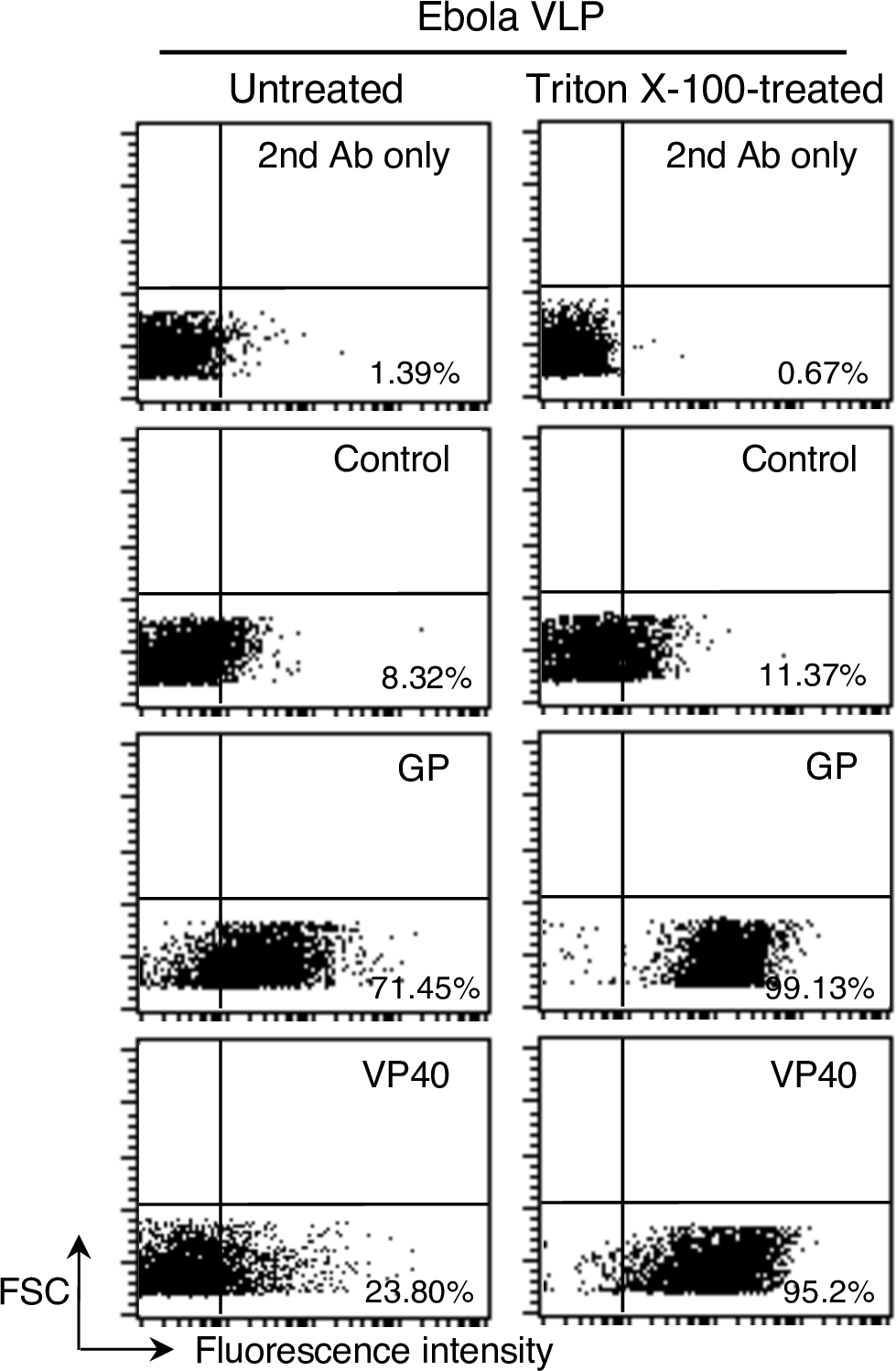

Supplement: S2 Fig — Ebola VLP-conjugated beads were incubated with or without 0.05% Triton X-100 in PBS containing 2% BSA for 10 min at room temperature. After being washed, the beads were incubated with rabbit polyclonal antibodies against EBOV GP, VP40, or LASV GPC, followed by incubation with Alexa Fluor 488-labeled secondary antibody. The binding of antibody to the beads was analyzed by flow cytometry. The percentages of the positive populations are indicated. 2nd Ab represents the beads that were not treated with primary antibody. X-axis: fluorescence intensity, Y-axis: forward scatter corner signals. The results are representative of three individual experiments. (TIFF) [file ppat.1006848.s002.tiff]

HEK293T

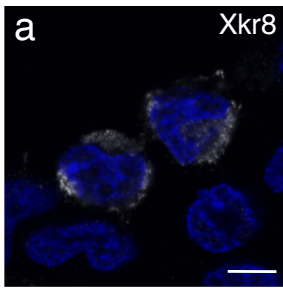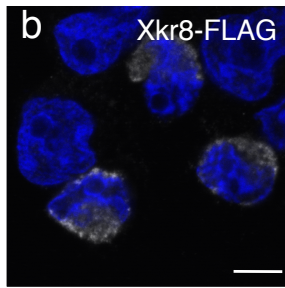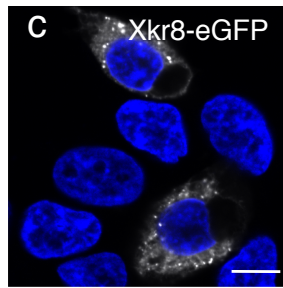

NU-GC-3

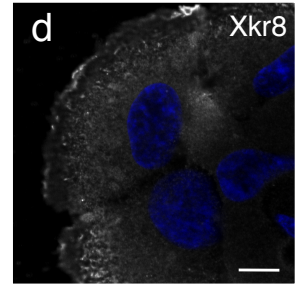

Scale bars: 10  $\mu$ m

Supplement: S3 Fig — HEK293T cells (a), HEK293T cells transiently expressing FLAG- (b) or GFP-tagged Xkr8 (c), and NU-GC-3 cells (d) grown on cover slips were fixed in 4% PFA followed by immunofluorescent staining with the rabbit polyclonal anti-Xkr8 antibody (a and d), or rabbit polyclonal anti-FLAG antibody (b) (Cell Signaling Technology). The intracellular distribution of endogenous or tagged Xkr8 was analyzed by using a confocal laser scanning microscope. The nuclei (blue) were counterstained with Hoechst 33342. Scale bars, 10 μm. (PDF) [file ppat.1006848.s003.pdf]

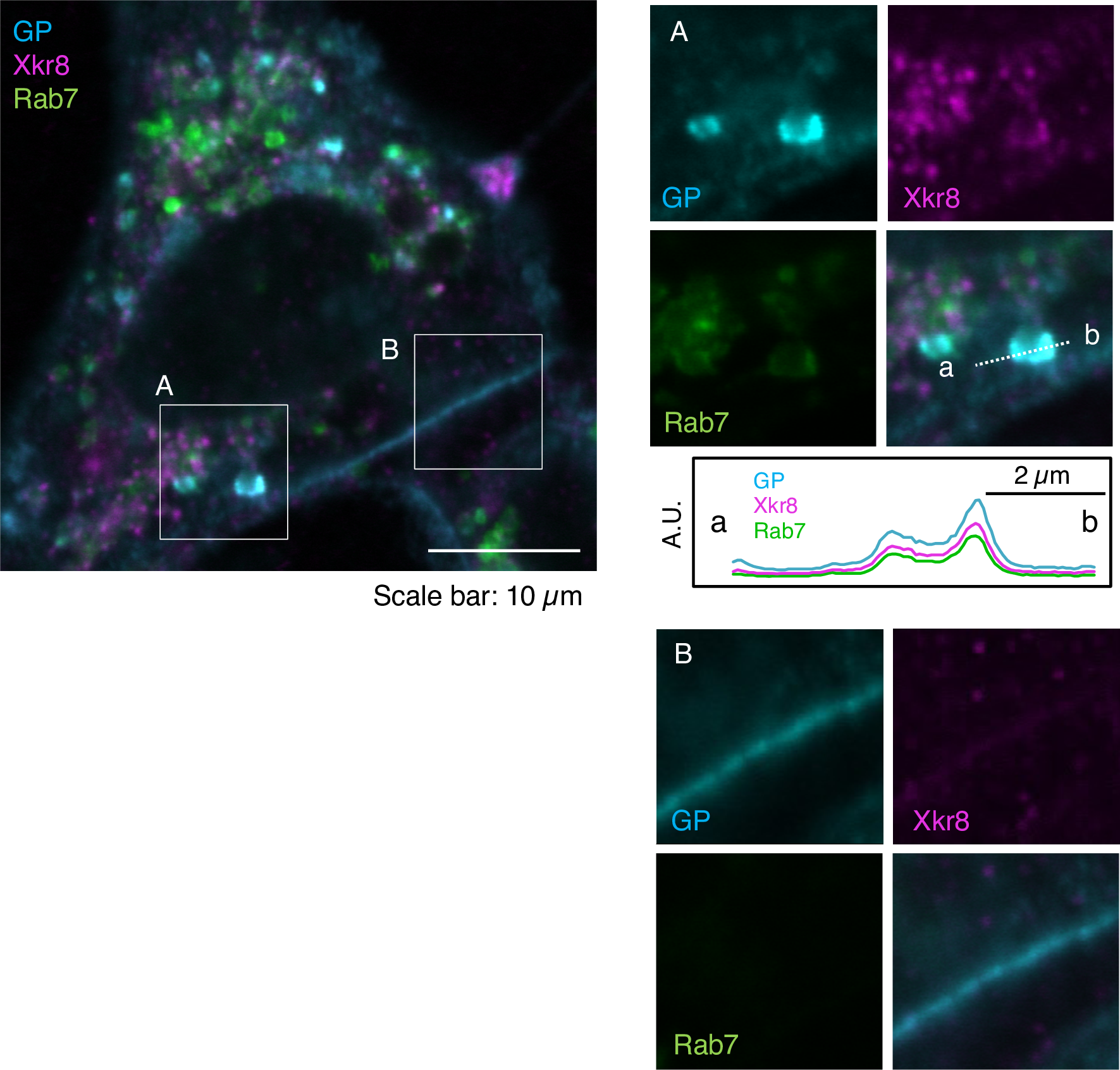

Supplement: S4 Fig — Vero-E6 cells stably expressing eGFP-Rab7 [4, 72] were transfected with an expression plasmid of EBOV GP. At 48 h.p.t., cells were fixed in 4% PFA and subjected to immunofluorescence staining with a rabbit anti-Xkr8 and anti-GP polyclonal antibodies. Insets show the boxed areas. eGFP-Rab7, GP, and Xkr8 are shown in green, cyan, and magenta, respectively. A and B represent boxed areas in the image. The plot indicates the relative fluorescence intensity of the individual channels along each of the corresponding lines. A.U.; arbitrary unit. Scale bar: 10 μm. (TIFF) [file ppat.1006848.s004.tiff]

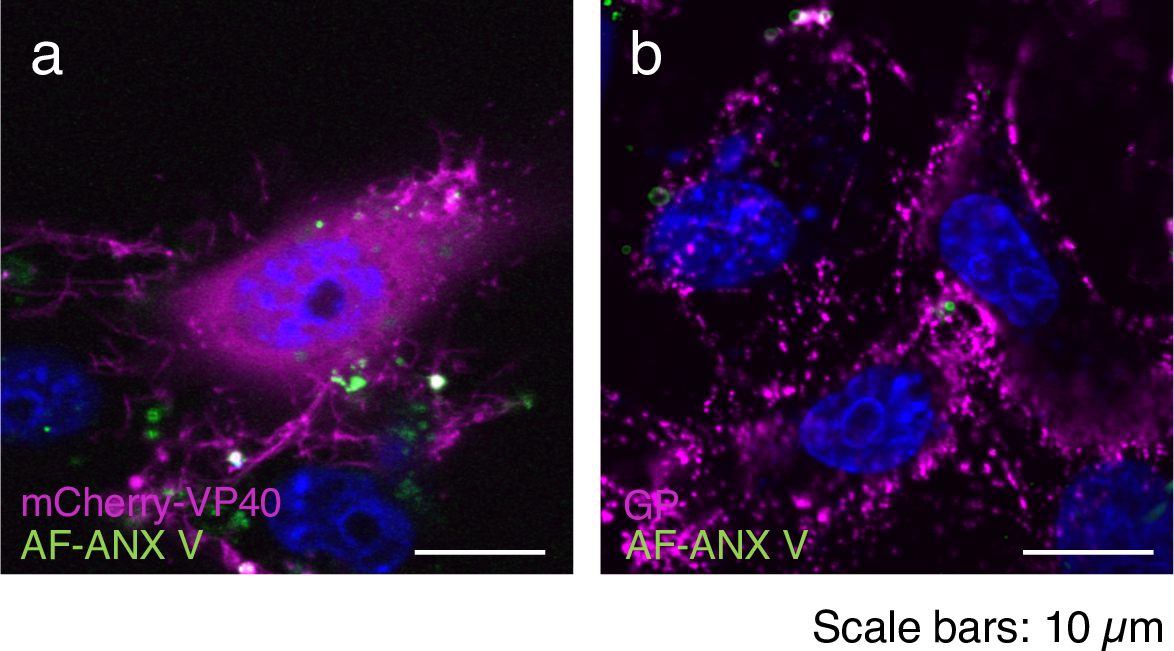

Supplement: S5 Fig — Vero-E6 cells grown on 35-mm glass bottom dishes were transfected with the expression plasmids of mCherry-VP40 and wtVP40 at a ratio of 1:5 (a), GP alone (b). At 72 h.p.t., the cells were harvested and followed by AF-ANX V staining. For detection of GP, the cells were incubated in the medium containing the anti-GP antibody, followed by incubation with Alexa Fluor 647-conjugated secondary antibody. After being washed with medium and ANX V binging buffer, the cells were treated with AF-ANX V. After washing again, the AF-ANX V signal (green) and EBOV proteins (magenta) were observed by using a confocal microscope. The nuclei (blue) were counterstained with Hoechst 33342. Scale bars : 10 μm. (TIFF) [file ppat.1006848.s005.tiff]
